# A User’s Guide for Phase Separation Assays with Purified Proteins

**DOI:** 10.1016/j.jmb.2018.06.038

**Published:** 2018-11-02

**Authors:** Simon Alberti, Shambaditya Saha, Jeffrey B. Woodruff, Titus M. Franzmann, Jie Wang, Anthony A. Hyman

**Affiliations:** Max Planck Institute of Molecular Cell Biology and Genetics, 01307 Dresden, Germany

**Keywords:** MBP, maltose-binding protein, GFP, green fluorescent protein, PCM, pericentriolar material, MAP, microtubule-associated protein, TEV, tobacco etch virus, RBD, RNA-binding domain, PLD, prion-like domain, FUS, Fused in Sarcoma, phase separation, membrane-less organelle, membrane-less compartment, prion-like protein, Sup35, low-complexity proteins, protein purification

## Abstract

The formation of membrane-less organelles and compartments by protein phase separation is an important way in which cells organize their cytoplasm and nucleoplasm. *In vitro* phase separation assays with purified proteins have become the standard way to investigate proteins that form membrane-less compartments. By now, various proteins have been purified and tested for their ability to phase separate and form liquid condensates *in vitro*. However, phase-separating proteins are often aggregation-prone and difficult to purify and handle. As a consequence, the results from phase separation assays often differ between labs and are not easily reproduced. Thus, there is an urgent need for high-quality proteins, standardized procedures, and generally agreed-upon practices for protein purification and conducting phase separation assays. This paper provides protocols for protein purification and guides the user through the practicalities of *in vitro* protein phase separation assays, including best-practice approaches and pitfalls to avoid. We believe that this compendium of protocols and practices will provide a useful resource for scientists studying the phase behavior of proteins.

## Highlights

• Membrane-less organelles form by phase separation.
• Membrane-less organelles can be reconstituted from minimal components.
• Phase-separating proteins are difficult to purify and handle.
• We provide guidelines and protocols for working with phase-separating proteins.

## Graphical Abstract

**Figure:**
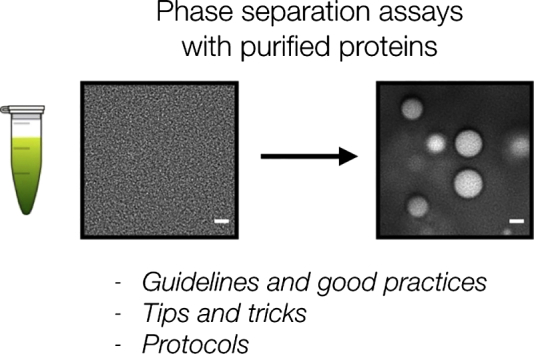

## Introduction

Cells contain many compartments that are not surrounded by membranes. These membrane-less compartments are thought to form by phase separation. Phase separation is a process in which a well-mixed solution of proteins and other macromolecules spontaneously separates into two phases, one phase that is enriched for the macromolecules and a surrounding phase that is depleted of the macromolecules [1], [2], [3]. Because the formed dense phase has a boundary that allows selective access of certain macromolecules, it can function as a compartment. Compartments that form by phase separation can be very dynamic. They often have properties of liquid droplets and rapidly exchange components with their surroundings. Maintenance of these droplet compartments requires a network of interactions, many of which are weak and transient [4], [5], [6], [7]. Proteins that mediate phase separation in the cellular environment often contain multiple self-interaction domains and have a high fraction of intrinsic disorder.

Various proteins have been purified and tested for their ability to phase separate and form liquid droplets. However, the results often differ between labs and are not easily reproduced. Thus, there is an urgent need for high-quality proteins, standardized procedures and generally agreed-upon good practices for performing phase separation assays. Another complicating factor is that many phase-separating proteins are very difficult to purify and handle. Currently, the most frequently used expression system for phase-separating proteins is bacteria. This is problematic because many phase-separating proteins have a complex domain organization, are modified with post-translational modifications, and are highly aggregation-prone in bacterial expression systems. For these reasons, we use insect cells to express phase-separating proteins, which reduces the aggregation propensity of these proteins and ensures that they receive the post-translational modifications that are required for normal protein activity and behavior. Indeed, several recent studies demonstrated that protein phase separation is regulated by post-translational modifications such as phosphorylation [5], [8]. In studies that investigate the role of post-translational modifications, it may be advantageous to express the protein in bacteria to obtain unmodified protein. We also have investigated the influence of pH, salt concentration, temperature, and solubilizing agents on reconstituting protein phase separation *in vitro*.

**Fig. 1:**
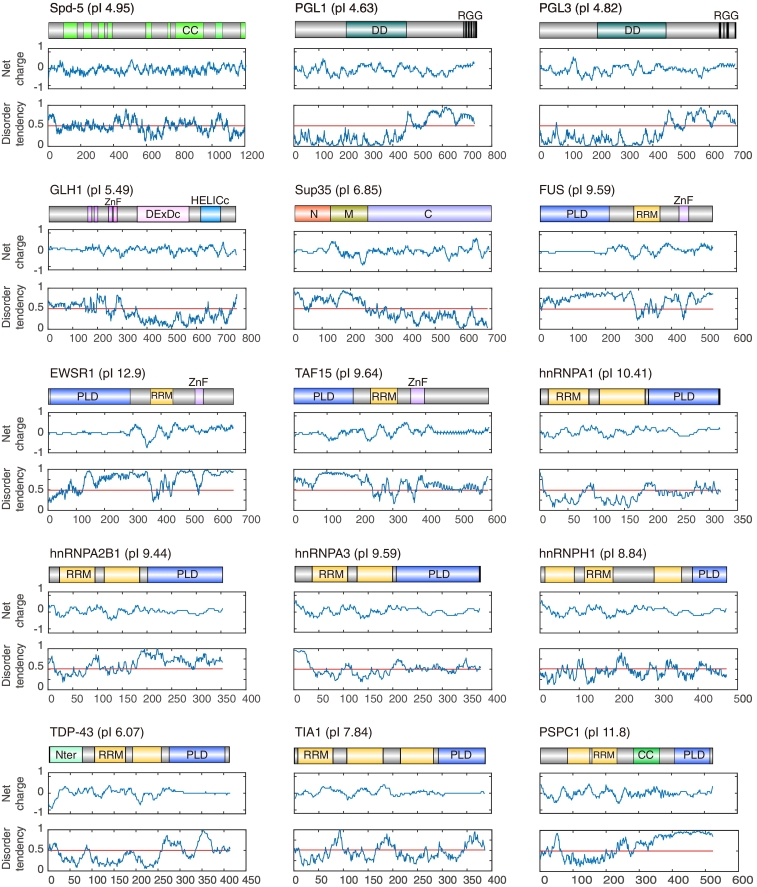
Domain structure, net charge, and disorder tendency of phase-separating proteins. The isoelectric point (pI) for each protein is shown after the protein name. CC, coiled-coil; Nter, N-terminal domain; DD, dimerization domain; ZnF, zinc finger; DEXDc, DEAD-like helicases superfamily; HELICc, helicase superfamily; N, N domain; M, middle domain; C, C domain; PLD, prion-like domain; RRM, RNA recognition motif. The net charge was plotted using a sliding window of 20 amino acids. The disorder tendency was predicted by IUPred (see Experimental procedures).

Here, we describe successful purification procedures for several proteins that we have studied in recent years. This includes centrosome proteins and P granule proteins from *Caenorhabditis elegans*, prion proteins from yeast, and prion-like proteins that cause diseases in humans (Fig. 1). We point out critical steps and caveats during the purification of these proteins, and we provide tips and tricks to prevent common problems. We hope that this compendium of protocols and observations provides a useful resource for scientists studying the phase behavior of proteins.

## General considerations

Before we begin to give a detailed description of the purification procedures of phase-separating proteins, we would like to mention some general considerations about how to handle them. Phase-separating proteins harbor architectural features, such as intrinsically disordered prion-like domains (PLDs) or coiled-coils, that make them self-interact and even aggregate at high concentrations, thus precluding purification [1], [9]. These proteins are also notoriously sticky and will bind to tubes and purification columns. Because of these complications, many standard purification strategies often do not work.

One common problem is that many phase-separating proteins are marginally soluble in common buffers. For this reason, we screen for conditions that enhance protein solubility and include solubilizing tags such as maltose-binding protein (MBP) or green fluorescent protein (GFP). Placing these tags adjacent to domains that drive phase separation (e.g., PLDs) can also interfere with self-assembly, thus enhancing solubility during purification.

Another common problem is that phase-separating proteins often stick non-specifically to surfaces, including many commercially available chromatography resins and tube walls. For sticky proteins, we recommend avoiding columns that require large bed volumes (e.g., gel filtration) or ion-exchange columns. Instead, one can use multi-step affinity chromatography with low-binding resins (e.g., Ni-NTA) and elution with protease cleavage. We often remove the tags at a late step of the purification procedure, thus allowing us to do downstream phase separation assays. We also recommend using low-binding tubes for protein storage and phase separation experiments.

It is also important to consider the salt concentration during protein purification. Globular proteins that do not phase separate are typically soluble in low-salt buffers and are stabilized at low temperatures around 4 °C. However, we have found that low-salt conditions and low temperature often promote phase separation of the proteins used in our studies. Thus, we recommend using high-salt buffers during protein purification and to perform protein purifications at room temperature whenever possible. However, this approach may not be useful for all proteins because low temperature and high salt can also sometimes induce phase separation. There are many instances in the literature where proteins that localize to membrane-less compartments were described as insoluble under standard purification conditions. We hypothesize that these occurrences of protein insolubility were often due to unwanted phase separation.

Phase separation of proteins is exquisitely sensitive to changes in physico-chemical conditions, such as concentration, pH, and buffer charge [10], [11]. High protein concentrations will drive phase separation. Thus, we recommend keeping the protein of interest dilute during purification. In addition, the buffer pH should be at least one unit away from the isoelectric point of the protein of interest to maximize solubility. Addition of charged amino acids, such as arginine, to the buffer can also improve protein solubility. Many proteins that we have worked with, such as Fused in Sarcoma (FUS), have a high content of arginine residues. These arginines can mediate homotypic interactions with aromatic amino acids and drive protein phase separation. Including arginine in the purification buffers interferes with such interactions and improves protein solubility.

To help with optimizing protein purification and visualizing protein phase separation, we recommend appending a GFP tag to the protein of interest. The GFP tag will not interfere with the phase behavior of the protein if the tag is put at the opposite end of the domain that drives protein phase separation behavior. It is important that the appended GFP or other fluorophore does not form dimers or higher-order oligomers. Normal versions of EGFP, which weakly dimerize, interfere with the normal phase behavior of our proteins. We therefore use a monomeric variant of GFP (A206K).

Finally, we have found that repeated freezing and thawing cycles strongly diminish protein quality, even more so than for globular proteins. We therefore advise freezing a purified protein in many small aliquots and to thaw a protein only once. For some proteins, a single freezing cycle can damage the protein. In these cases, we recommend using the freshly purified protein for phase separation assays right after purification without freezing.

### Production of baculovirus proteins in insect cells

To produce protein from insect cells, we first generate recombinant baculovirus that will infect the cells and express the target protein of interest. This involves transforming insect cells with a viral genome (bacmid) and subsequently amplifying virus titers through several rounds of infection. We summarize the general steps for producing viruses below. Note: we use an in-house system (a detailed description and protocol will be published elsewhere), but commercially available systems are currently available (e.g., Bac-2-Bac).

1) The genes encoding the target protein of interest are cloned into a baculovirus shuttle vector.
2) The recombinant shuttle vectors are co-transfected with a replication-deficient and modified BV (baculovirus) genome in insect cells.
3) Homologous recombination between overlapping regions of the shuttle vector and bacmid yields a baculovirus genome that is competent to replicate in insect cells and thus produces recombinant baculovirus (P1 stock).
4) The viruses contained in the P1 supernatant are used to infect 50 ml SF9 ESF cells and obtain P2 stocks. A high titer P2 supernatant is collected and stored for later use.
5) The P2 virus stock is used to infect cells for protein expression and harvest. Typically, we first perform a small-scale purification with cells harvested from 2 ml liquid cultures to test for expression level, solubility, the binding efficiency onto the affinity resin, purity, and the efficiency of tag cleavage by protease. Proteins that successfully go through all of these steps are then submitted to a large-scale purification, which is normally done with the harvested cells obtained from 500 ml liquid cultures.

### Protein purification of *C. elegans* P granule proteins

#### Introduction

P granules are membrane-less organelles containing protein and RNA and are required for normal germline development and fertility in *C. elegans* [12], [13], [14], [15]. P granules belong to a family of conserved “germ granule” organelles found in the germline of sexually reproducing animals [12]. More than 40 proteins are known to concentrate in P granules, but only 7 of these proteins are present in P granules throughout the lifetime of *C. elegans*: PGL-1, PGL-3, GLH-1, GLH-2, GLH-3, GLH-4, and DEPS-1 [16], [17], [18], [19], [20], [21]. The other proteins localize to P granules transiently. Since P granules are known to be liquid-like condensates that form via phase separation [7], [22], we tested if some of the non-transient P granule proteins (PGL-1, PGL-3, or GLH-1) have the ability to phase separate *in vitro*. Among the three proteins, only PGL-3 was able to phase separate into a P granule-like condensate *in vitro* in the absence of crowding agents, suggesting that PGL-3 scaffolds the assembly of P granules *in vivo* [7].

#### Experimental approach

A single strategy for expression and purification worked well for PGL-3, PGL-1, and GLH-1. A detailed protocol for purification of PGL-3 is available elsewhere [7]. Briefly, recombinant baculovirus expressing these three P granule proteins was used to infect SF9 insect cells. Recombinant P granule proteins produced in the insect cells were purified using a combination of Ni-NTA affinity, ion-exchange, and size-exclusion chromatography. To allow for Ni-NTA affinity chromatography, these proteins were tagged at the C-terminus with a 6 ×-Histidine tag (6 × His), followed by monomeric-enhanced GFP (mEGFP). A linker containing a proteolytic cleavage site [e.g., PreScission or tobacco etch virus (TEV)] was added between the protein of interest and the 6 × His-mEGFP tag to allow for removal of the tag, if desired, before storage.

#### Specific considerations

We list a few strategies below that were helpful while purifying and storing P granule proteins.

1) Addition of l-arginine increased the solubility of recombinant P granule proteins in insect cell lysates, boosting yield of purified protein.
2) Ideally, purification should be carried out under conditions that disallow phase separation of the protein of interest. To find conditions that disallow phase separation, one or more parameters could be varied: ionic strength of buffer, pH, temperature, concentration of the phase-separating protein(s) itself and those of other coexisting macromolecules (like proteins and RNA) or small molecules (like adenosine triphosphate). Sometimes, however, it is difficult to avoid protein phase separation during purification.
3) In cases where phase separation of the protein of interest into highly viscous or solid-like condensates cannot be avoided during purification, a batch-mode of purification works better than using prepacked columns. For instance, in preparation for anion-exchange chromatography, the protein PGL-3 must be dialyzed into a low-salt buffer. This leads to phase separation of PGL-3 into liquid-like condensates. We found that pure PGL-3 protein can be obtained using a prepacked anion-exchange column (HiTrap Q column; GE Healthcare). However, complications occur when the same strategy is applied to purifying a mutant PGL-3 protein which phase separates into highly viscous or solid-like condensates. In this case, the mutant PGL-3 protein is found to associate with the HiTrap Q column tightly, such that the protein cannot be eluted even at > 2 M KCl conditions, resulting in failed purification. It usually takes high concentrations of denaturant like urea or guanidine hydrochloride to dissociate the mutant PGL-3 protein from the column. We circumvented this problem by using batch-mode anion-exchange chromatography (with Q Sepharose beads) to purify the mutant PGL-3 protein. The protein binding and wash steps were carried out under conditions where the Q sepharose beads were far less tightly packed in space compared to that in prepacked columns (for instance, in 50 ml solutions containing less than 5 ml of Q sepharose beads). Low-speed centrifugation (around 1000 *g*) was used to separate the beads from the rest of the solution at the end of each step. Using batch-mode purification allowed mutant PGL-3 protein to unbind the beads and elute into a high-salt buffer.
4) It is useful to monitor the “phase separation status” of the protein of interest throughout the purification process. One strategy is to purify the protein of interest tagged to a fluorescent protein. This provides two advantages. First, the fluorescent protein can be tracked readily by visual inspection using the naked eye. Second, it also makes it easy to monitor the phase separation status of the protein of interest using epifluorescence microscopy.
5) Following purification, we store the PGL-3 protein in a non-phase separated form under high salt conditions. We trigger phase separation by dilution to physiological salt levels only before performing an assay. The P granule proteins that do not phase separate on their own (PGL-1 and GLH-1) can be stored in buffers containing physiological salt concentration.

### Purification of *C. elegans* centrosome proteins

#### Introduction

Centrosomes are membrane-less organelles that nucleate and organize hundreds to thousands of microtubules needed for chromosome segregation during mitosis in animal cells. Centrosomes are composed of structured centrioles that organize a more massive but less ordered layer of protein called pericentriolar material (PCM), which serves as the nucleation bed for microtubules [23]. The PCM scaffold is built through the self-assembly of elongated proteins rich in coiled-coil domains, such as SPD-2 (most eukaryotes), Centrosomin (flies), Cdk5Rap2 (vertebrates), or SPD-5 (*C. elegans*) [24]. This scaffold then becomes competent to nucleate microtubules by recruiting tubulin and globular enzymes that catalyze microtubule nucleation and growth, called microtubule-associated protein (“MAPs”). Mitotic kinases, such as Polo-like kinase and Aurora A, enable PCM assembly and activity through phosphorylation of scaffold proteins and the MAPs.

#### Experimental approach

We recently reconstituted *C. elegans* PCM assembly and microtubule nucleation using eight purified proteins: two scaffold proteins (SPD-5 and SPD-2), three MAPs (ZYG-9, TPXL-1, and TAC-1), the Polo-like Kinase PLK-1, and alpha- and beta-tubulin [25], [26]. Native alpha/beta-tubulin dimers were purified using standard protocols [27]. All PCM proteins were generated from recombinant baculoviruses used to infect SF9 insect cells. Baculoviral expression was required to produce fully active proteins; bacterial expression produced soluble but inactive target protein. Standard approaches were used to purify the MAPs and PLK-1, while special considerations were required for the scaffold proteins. A detailed protocol can be found elsewhere [26].

All MAPs and PLK-1 were appended with a 6 ×-Histidine tag (6 × His) connected with a linker containing a proteolytic cleavage site (e.g., PreScission or TEV). To release the proteins, SF9 cells were lysed using dounce homogenization or high pressure-induced cavitation (Emulsiflex). In our hands, use of the Emulsiflex achieved higher yields of soluble protein. The lysate was centrifuged, and the high-speed supernatant was passed over Ni-NTA resin. After elution from the Ni-NTA column, the 6 × His tag was cleaved off, and then the target proteins were further purified by size-exclusion chromatography and ion-exchange chromatography.

#### Specific considerations

We found that the scaffold proteins SPD-5 (135 kDa, nine predicted coiled-coil domains) and SPD-2 (92 kDa, one predicted coiled-coil domain) were prone to degradation in the host insect cells and would interact non-specifically with many purification resins. Thus, we could not use a standard three-step approach as outlined above. The following considerations improved protein expression, purity, and yield:

1) Tag the N-terminus of the target protein with MBP to reduce degradation. We found that SPD-5 is degraded inside insect cells, prior to lysis. Appending a MBP tag to the N-terminus prevents this degradation. This effect has been reported for other proteins elsewhere (genescript.com).
2) Use Ni-NTA resins, but avoid amylose, sepharose, and sephadex. We had great success with specific binding of 6 × His-tagged SPD-5 and SPD-2 to Ni-NTA resin and subsequent elution. However, these proteins would strongly bind to amylose, sepharose, and sephadex resins in a non-specific manner. After binding MBP-tagged SPD-5 to amylose resin, we typically recovered < 10% of the input protein after elution with 15 mM maltose. Passing SPD-5 or SPD-2 over gel filtration columns (e.g., Superdex series columns, which contain sephadex resin) resulted in near 100% loss. Similar results were obtained when passing these proteins over ion-exchange resin (Q or SP sepharose). Thus, we recommend using affinity chromatography with Ni-NTA resin and avoiding gel filtration and ion exchange.
3) Use proteolytic cleavage to elute target proteins from affinity resins. Proteolytic elution enhances purity of the final product. For example, many native insect cell proteins will bind to Ni-NTA resin in addition to the 6 × His-tagged target protein. A standard elution with imidazole will release all bound proteins. However, elution by proteolytic cleavage will release only the target protein. We typically insert cleavage sites in linker regions connecting the target protein to purification tags. For cleavage, we favor PreScission protease (recognition site: LEVLFQ/GP) as it works efficiently at 4 °C. We also use TEV protease (ENLYFQ/G).
4) For protocol optimization, use fluorescently labeled target protein. When expressing and purifying a target protein for the first time, we recommend using a GFP- or mCherry-tagged version. This allows the user to quickly and easily optimize each step (e.g., expression, binding to resin, minimizing non-specific interactions, elution) using a fluorescent microscope. This saves time and material compared to analyzing coomassie-stained SDS-PAGE gels.
5) Screen for buffers that prevent self-assembly and/or non-specific interactions with chromatography resins. Centrosome scaffold proteins are remarkable for their ability to self-assemble and recruit numerous proteins. That is their job in the cell. This means that they will also self-assemble and bind unwantedly to proteins and purification resins, which can negatively impact purity and yield. We recommend using a visual screen using fluorescence microscopy (see above) to test different buffer conditions to prevent self-assembly and binding to a general resin. Buffer conditions include salt concentration, pH, type of non-ionic detergent type, and solubilizing additives (arginine, glutamate, glycerol, < 10% ethanol). We typically use buffers containing 25 mM Hepes (pH 7.4), 500 mM NaCl, 0.1% Chaps, and 1% glycerol.

### Protein purification of the yeast prion protein Sup35

#### Introduction

Sup35 is a highly conserved protein required for the termination of protein synthesis at the ribosome. The Sup35 protein from budding yeast has been studied extensively over the last 25 years. It has an intrinsically disordered N-terminal domain (called N domain) that is enriched for polar amino acids followed by a highly charged middle domain (called M domain). The C-terminal domain is folded and executes the catalytic function of the protein. Sup35 has become a model for understanding amyloid-like aggregation and the biology of heritable protein aggregates called prions. Importantly, discoveries made with Sup35 have substantially contributed to our understanding of human diseases that are caused by infectious protein aggregates such as prion diseases.

Because purified Sup35 has been under investigation for a very long time, we take a little more space here to describe its biochemistry. Early on it became clear that the N-terminal part of Sup35 has special properties. It was this part of the protein that made it challenging to purify Sup35: the isolated N-terminal domain aggregated readily during purification. It was clear that this must have to do with the unusual amino acid sequence of this domain. The N domain is largely depleted of hydrophobic residues but highly enriched for polar residues such as asparagine, glutamine, serine, and tyrosine [28]. Because of this amino acid composition, the N domain of Sup35 does not adopt a stable structure and thus is disordered under physiological conditions [29]. However, under the right conditions, it can assemble into highly ordered amyloid aggregates [30], [31]. The N-terminal region was later defined as “prion domain” because it carries all the information for protein aggregation and prion behavior. As the founding member of the class of yeast prion proteins, Sup35 gave rise to a whole class of sequence-related proteins with PLDs [28], [32], [33]. Like Sup35, other prion-like proteins are intrinsically disordered and prone to aggregate during purification.

Only few attempts have been made to purify Sup35 under native conditions [30], [31], [34]. Ion-exchange chromatography was often the method of choice for native Sup35 purifications, but this required the use of low salt concentrations. This was a problem because low salt concentrations also increased the risk of Sup35 precipitation. Sup35 also tended to precipitate at low temperatures [30], [31]. This was also problematic because cooling was and still is common practice to stabilize proteins against aggregation and degradation.

Because of the high propensity of Sup35 to precipitate and the intrinsically disordered nature of the N domain, non-native purification strategies were developed [30], [35]. Most commonly, Sup35 fragments were expressed in *Escherichia coli*. During lysis of the bacteria and throughout the purification procedure, high concentrations of denaturant (commonly 6–8 M urea) were used to increase protein solubility and prevent the prion domain from aggregating. Afterward the purified protein was often precipitated with methanol for long-term storage. Importantly, diluting the denatured protein into a physiological buffer (devoid of denaturant and organic solvents) caused aggregation of the prion domain of Sup35 into amyloid-like fibers, serving as a further justification for the use of denaturing purification procedures [36]. As a consequence, denaturing purifications became the standard procedure for obtaining Sup35 protein. Given the success of the non-native purification procedure, much research focused on the disordered regions (NM), but not on the full-length protein in which the disordered regions are linked to the folded C-terminal domain.

We recently established a new protocol for purifying Sup35 under native conditions from insect cells [37]. This approach requires the use of a high-salt buffer to keep the protein soluble during the purification procedure and thus allows comparison of full-length protein to N-terminal fragments of Sup35 (NM) that were purified under native conditions. Using this approach, we find that Sup35 has a tendency to phase separate into liquid droplets that subsequently turn into gel condensates. Interestingly, when purified under native conditions, the N-terminal regions of Sup35 had a higher tendency to form aggregates compared to the full-length protein (which would rather phase separate into liquid droplets). Likewise, the C-terminal domain by itself aggregated in the absence of the disordered regions. This suggests an interaction between the folded C-terminal domain and the disordered N-terminal region that is required to keep the full-length Sup35 protein in a soluble state.

Further experiments *in vivo* revealed that the ability of Sup35 to form gel condensates is a conserved and physiological function of the prion domain of Sup35 that protects the protein from stress-induced damage [37]. These observations suggest that what was previously described as precipitation or aggregation behavior is a natural property of the Sup35 protein that has been shaped by evolution to make the protein more resistant to stress. These observations helped us develop a new perspective on how to purify Sup35 and related prion-like proteins, and they made clear that common lab practices for protein purification such as cooling might be contraindicated for this particular group of proteins.

#### Experimental approach

**Fig. 2:**
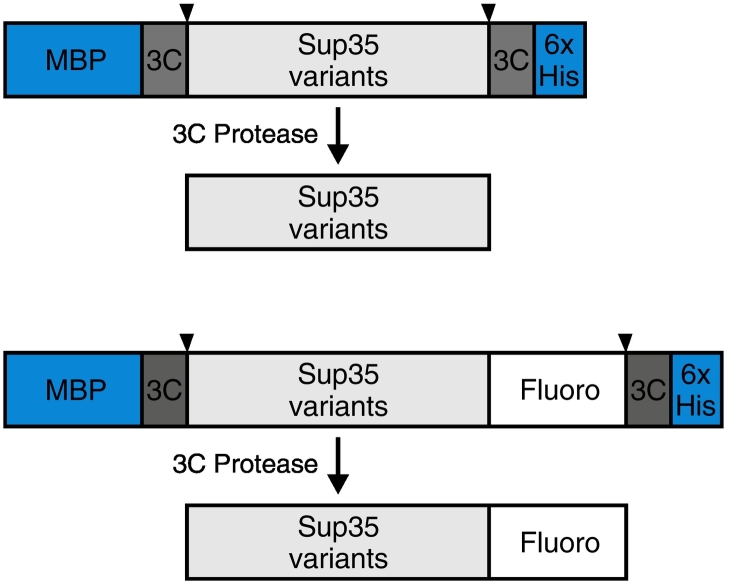
Constructs used to express Sup35. “3C” denotes a 3C PreScission protease cleavage site, “6 × His Tag” is a 6 × His tag for affinity purification, and “MBP” stands for maltose-binding protein that was used as an affinity tag and for solubilizing the fusion protein. Sup35 was also expressed as a fluorescence proteins fusion (Fluoro), such as monomeric sfGFP, TagRFP, or SnapTag.

To purify Sup35 from insect cells, we generated the following constructs (see Fig. 2). Use of these types of expression constructs was guided by the following considerations:

• The 6 × His tag can be used as affinity purification; however, we found that binding to a HisTrap column was poor, suggesting that the 6 × His tag may not be optimally exposed.
• The MBP tag improved the expression of Sup35 protein.
• Positioning the MBP tag before the prion domain of Sup35 suppressed premature phase separation and aggregation during purification. The MBP tag was cleaved off in the late steps of purification.
• The GFP tag was used to visualize Sup35 under the microscope. GFP fluorescence was also used to monitor the Sup35 behavior during every step of protein expression and purification.
• The GFP tag was placed behind the C-terminal domain of Sup35 far away from the prion domain. The tag in this position did not alter the phase behavior significantly.
• The PreScission protease sites were introduced between the MBP tag and 6 × His tag to allow for removal of the tags.

To produce wild-type and variant full-length Sup35, we infected SF9 insect cells with recombinant baculovirus expressing Sup35 fused to MBP (N-terminus) and 6 × His (C-terminus). The same approach was used to express fragments of Sup35, such as the disordered regions (NM), or a construct containing only the M and C domain (MC). Briefly, purification involved the following steps:

1) SF9 cells were infected 1:100 with high titer P2 baculovirus suspension and incubated at 27 °C for 72 h.
2) Cells were then harvested by centrifugation, washed once with buffer A [50 mM Tris–HCl, 1 M KCl, 2 mM EDTA, 1 mM DTT (pH 7.5)] and lysed in buffer A supplemented with cOmplet Protease Inhibitor Cocktail (Roche) using an Emulsiflex C5 (Avestin).
3) The lysate was cleared by centrifugation [20,000 rpm, JA-25.50 rotor (Beckman Coulter), 60 min, 4 °C].
4) The cleared supernatant was passed over amylose resin (NEB) and washed with 20 column volumes of buffer A.
5) Bound Sup35 was eluted with 20 mM maltose in buffer A.
6) Samples were pooled and GST-tagged 3C PreScission protease was added to cleave off the MBP- and 6 × His-tag during dialysis against buffer A overnight at 4 °C.
7) The sample was cleared by centrifugation and subjected to size-exclusion chromatography using a Superdex-200 26/60 column (GE Healthcare Life Sciences) equilibrated with buffer A running on a BioCad 60 (Applied Biosystems) at RT.
8) The pooled samples were concentrated and frozen in liquid nitrogen.

#### Specific considerations

1) Throughout the purification, Sup35 should be kept in a diluted state and should not be concentrated extensively. Concentrated protein solutions of Sup35 promoted premature phase separation as well as unwanted aggregation.
2) An MBP–Sup35 fusion protein was soluble in cells and after cell lysis after clearing the lysate. The MBP–Sup35 fusion protein bound weakly, yet specifically to amylose resin. MBP–Sup35 bound less effectively to dextrin resin packed MBPTrap HP columns (GE) compared to amylose resin (NEB). Likewise, the fusion protein also bound only weakly to nickel-chelating resins. In this case, the purity was substantially less compared to MBP.
3) When purifying in batch mode, we avoided inversion of the Sup35-bound resin and shear stress.
4) To avoid leakage of weakly bound Sup35 from the column, we omitted maltose in the binding and wash steps.
5) A fraction of full-length Sup35 was found in the flow-through fraction. This pool did not bind any of the tested MBP and Ni-NTA resins.
6) We advise taking advantage of larger columns in order to load larger volumes of less concentrated samples (e.g., for gel filtration).
7) The protein concentration should remain low (typically below 10 μM) during dialysis at low temperature. We found that Sup35 is prone to premature phase separation and unwanted aggregation during these conditions.
8) Steps such as size-exclusion chromatography or sample storage may require that the sample is concentrated. Prior to any concentration step, we subjected the protein to a high-speed spin at RT to remove possible aggregates.
9) We generally kept an aliquot at room temperature for 1 day and tested for degradation and aggregation using SDS-PAGE analysis, UV VIS spectroscopy, or fluorescence microscopy.

**Fig. 3:**
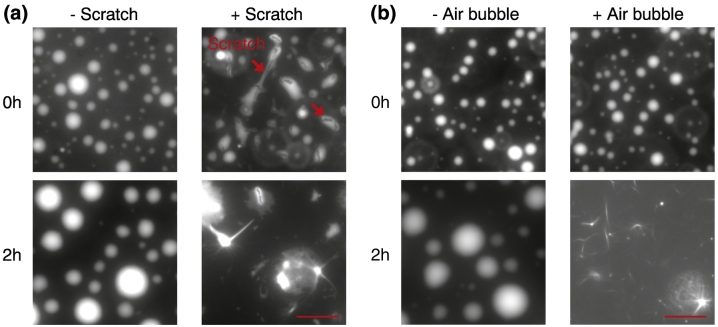
Scratches or air bubbles promote a liquid-to-solid transition of FUS. (a) Effect of scratches on liquid-to-solid transitions of FUS. The bar represents 10 μm. (b) Effect of air bubbles on liquid-to-solid transition of FUS. The bar represents 10 μm.

Purified Sup35 shows a rich phase behavior and is sensitive to pH, salt, and temperature changes. We performed phase separation assays with Sup35 in the following way:

1) Protein-rich droplets of Sup35 were formed by dilution of freshly purified and concentrated Sup35 into 20 mM Pipes, 2% polyethylene glycol (PEG; MW = 20,000). Alternatively, Sup35 protein was diluted from a frozen stock solution.
2) The pH was adjusted with NaOH and the respective pH of the phase separation buffer was tested at protein concentrations ranging from 0.1–20 μM. This is important because phase separation of Sup35 is strongly dependent on the pH of the solution.
3) Samples were mixed in low-binding PCR vials and imaged on coverslips passivated with PEG–silane (see Appendix A) or in 384 low-binding multi-well microscopy plates (Greiner Bio-One).
4) Liquid-like condensates of Sup35 are pH, salt, and temperature dependent. Controlling these parameters was important to obtain reproducible results.
5) Phase separation into liquid condensates was dependent on the purity of the protein preparation. Contaminants impair this process and cause aggregation.
6) Liquid-like condensates solidified with time into gel condensates. The gelation process is pH and salt dependent.
7) The protein concentration was determined before and after storage. Condensate formation was found to be temperature dependent and during condensation Sup35 sometimes stuck to the vial resulting in a change in protein concentration. Therefore, low-binding vials should always be used for phase separation assays.
8) The sample should never be mixed with a pipette. Samples in tubes should be mixed by flicking the tube and samples in plates by placing the plate on a shaker. We also avoided air bubbles (water/air interfaces cause aggregation; see Fig. 3).

### Protein purification of human prion-like proteins

#### Introduction

Human prion-like RNA-binding proteins are an abundant group of proteins that have been implicated in the formation of RNP granules [6], [38], [39]; they have also been associated with age-related neurodegenerative diseases such as amyotrophic lateral sclerosis and frontotemporal dementia [6], [38], [40], [41], [42]. Prion-like RNA-binding proteins contain domains of low-sequence complexity called PLDs and domains that bind RNA (RNA-binding domain). The PLD has a sequence composition similar to that of the Sup35 prion domain. Consequently, prion-like proteins show similar phase behavior to Sup35, with a strong tendency to phase separate and form aggregates in a manner that depends on the solution pH, salt concentration or temperature. Depending on the particular protein, the PLD can be either in the N-terminal or in the C-terminal part of the protein. Typically, the entire PLD region and a large portion of the RNA-binding domain region are intrinsically disordered.

#### Experimental approach

**Fig. 4:**
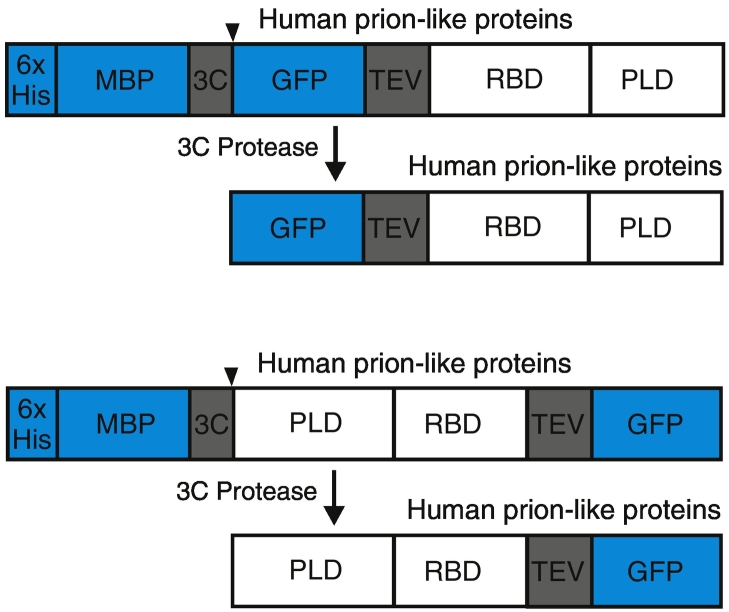
Constructs used for expression of human prion-like proteins. “3C” represents a PreScission protease cleavage site, and “TEV” represents a TEV protease cleavage site. The MBP tag was used to improve the expression and solubility of the prion-like proteins. To increase the binding to the affinity column, the 6 × His tag was used. After cleavage by the PreScission protease, one terminal of the PLD was released from the tag. It has been noticed that for some prion-like proteins, such as FET proteins, phase separation can be suppressed when one terminal of the PLD is not released.

We have recently purified more than two dozen human prion-like proteins to investigate their involvement in RNP granule formation and disease [43]. Our most frequently used constructs for expression of these proteins are shown in Fig. 4.

When working with prion-like proteins, we advise not permanently modifying regions in or near PLDs as this impairs phase separation. Therefore, for proteins with the PLDs at the N-terminus, the 6 × His–MBP–3C–Target protein–TEV–GFP construct was used; for proteins with the PLD at the C-terminus, a construct encoding 6 × His–MBP–3C–GFP–TEV–Target protein was used. PreScission protease was used in the late steps of purification to cleave off the 6 × His–MBP tag, leaving the PLDs unencumbered. A typical protein purification procedure involved the following steps:

1) SF9 insect cell pellets were resuspended in lysis buffer containing 50 mM Tris–HCl (pH 7.4), 1 M KCl, 5% glycerol, and 10 mM imidazole. Protease inhibitors (Calbiochem, Lot 2829749, 1 mM PMSF, 100 μM AEBSF, 0.08 μM Aprotinin, 5 μM Bestatin, 1.5 μM E-64, 2 μM Leupeptin, and 1 μM Pepstatin A) were then added.
2) The cells were lysed by sonication and the crude lysate was clarified by centrifugation at 13,000 rpm for 20 minutes.
3) The supernatant was incubated with Ni-NTA agarose resin (QIAGEN, Cat. No. 30230), which was pre-equilibrated with the lysis buffer.
4) The agarose beads were collected by centrifugation for 2 min at 1500 rpm and were resuspended into the lysis buffer by gently mixing in order to wash out the non-specifically bound proteins.
5) The washed beads were further collected by centrifugation for 2 min at 1500 rpm and were resuspended into the lysis buffer again. The mixture was transferred to the gravity columns [Micro Bio-Spin Chromatography Columns (Bio-Rad, 732-6204) or Econo-Pac Chromatography Columns (Bio-Rad, 7321010)] and the beads were further washed with 2 column volumes of lysis buffer.
6) The proteins were then eluted with buffer containing 50 mM Tris–HCl (pH 7.4), 1 M KCl, 5% glycerol, and 500 mM imidazole.
7) PreScission protease was added to the eluted protein at a ratio of 1:50 to cleave off the 6 × His–MBP tag. The mixture was incubated at room temperature for 1 h (small-scale purification) or 4 h (large-scale purification).
8) For large-scale purifications, the cleaved protein sample was incubated with amylose resin (Biolabs) to remove the 6 × His–MBP tag. The flow-through was further purified through gel filtration chromatography equilibrated with the storage buffer containing 50 mM Tris–HCl, 500 mM KCl, 1 mM DTT, and 5% glycerol. Peak fractions were pooled, subsequently concentrated, and aliquoted in PCR tubes, flash-frozen in liquid nitrogen, and stored at − 80 °C.

#### Specific considerations

1) We found that a small-scale purification can be done in a high throughput way by taking advantage of a 24-well Blocks RB plate (Qiagen). In this case, 24 different constructs can be tested simultaneously in one plate, with 2 ml cells cultured in each well. After culturing, further purification can be performed with a His SpinTrap column (GE Healthcare) in a high-throughput manner.
2) The protein should be pure enough to avoid formation of what we call “sticky balls” in phase separation assays. Sticky balls are droplets that arise from impurities and they show no sign of fusion; that is, the resulting droplets just stick to each other. An additional affinity purification step using the MBP tag or ion-exchange chromatography should be included in order to remove such impurities.
3) The removal of the 6 × His–MBP tag can lead to unwanted phase separation. We noticed that high concentrations of KCl or imidazole can improve the solubility of certain prion-like proteins by suppressing phase separation. KCl (1 M) and imidazole (500 mM) are generally required to suppress unwanted phase separation induced by protein cleavage. We also found that adding l-arginine promotes protein solubility and dissolves droplets [44].
4) Phase separation was sometimes induced through low temperatures. To avoid temperature-induced phase separation during purification, all the steps starting from protease treatment should be done at room temperature.
5) After cleavage and concentration, the protein should always be subjected to gel filtration as soon as possible. Long-term incubation of the concentrated solution after cleavage can promote protein aggregation.
6) Certain prion-like RBPs have a tendency to form fibers during the concentrating steps; thus, the final concentration for those proteins should be in a range optimal for solubility. For example, the highest concentration for hnRNPA2B1 or hnRNPA1b (the long isoform of hnRNPA1) can only be around ~ 200 μM.

### Phase separation assays

Our standard way of performing phase separation assays with prion-like proteins and other proteins was to dilute the purified protein from a high-salt, high-protein concentration solution into a buffer that recapitulates physiological salt conditions. To induce phase separation at low-salt conditions, proteins were diluted into the corresponding experimental buffers at various concentrations in a total solution volume of 20 μl. For droplet formation in the presence of crowding agent, proteins at the indicated concentrations were mixed with 10% Dextran in buffer containing 25 mM Tris–HCl (pH 7.4), 150 mM KCl, 2.5% glycerol, and 0.5 mM DTT. The samples were then transferred into the 384-well non-binding microplates (Greiner bio-one). Phase separation was determined by fluorescence microscopy. Images were usually taken after all the droplets have settled down to the bottom of the plate. To quantify phase separation, we plotted the amount of the condensed protein *versus* protein concentration. The amount of the condensed protein is defined by the ratio of I_droplet_ to I_outside_, where Idroplet is the integrated intensity inside the droplets and Ioutside is the integrated intensity outside the droplets (see Experimental procedures).

In a standard protein phase separation assay, the samples were thawed by incubating them at room temperature for 5 min before use. Depending on the turbidity of the thawed solution, different methods were adopted to further treat the protein samples and determine the concentration. If the protein solution appeared transparent, the sample was spun down at 13,000 rpm for 5 min, and the supernatant was used for determining the concentration with NanoDrop ND-1000 spectrophotometer (Thermo Scientific). In cases where the protein solution was turbid after thawing, the sample was first checked under the microscope to determine whether the protein had formed irregular aggregates. The sample was then centrifuged at 13,000 rpm for 5 min to remove the aggregates as much as possible. The concentration of the supernatant was determined and it was then used in phase separation assays. If the thawed protein solution contained liquid droplets, a small aliquot of the protein sample was taken and diluted until the solution was no longer turbid. The resulting solution was then used to determine the protein concentration. The concentration of the stock protein solution was then calculated from the concentration of the diluted sample. The original stock solution was used for phase separation assays.

To perform phase separation assays with these proteins, we followed the following steps:

1) We found that it was better to add the protein first, and then induce phase separation by adding phase separation buffer. This ensured that the protein was well mixed in the buffer. Adding the protein into the phase separation buffer could induce aggregation, probably due to a slower mixing. Generally, the sample should be mixed gently and air bubbles should be avoided during every step of sample preparation because fibril formation may be nucleated at air-water interfaces (see Fig. 3).
2) It is desirable to use an inverted microscope rather than an upright one to image phase-separated condensates since these condensates tend to settle by gravity. Depending on the density of the surrounding medium, the condensates could settle down at very slow or fast rates. While slowly sedimenting condensates could be imaged in suspension, fast-sedimenting condensates were imaged once they have settled down on the surface of the cover glass.
3) We found that coating the cover glass or bottom of the plate with a supported lipid bilayer or PEG-silane (see Appendix A) preserved the material properties of condensates for sufficiently long to allow imaging (detailed protocol for coating cover-glass provided in Appendix A).
4) When using 384-well non-binding microplates, the bottom of the plate should not be scratched. Scratched surface induced changes in material properties of the liquid droplets (see Fig. 3).
5) In fluorescence-based assays to monitor the dynamics of phase separated condensates, it is advisable to use a predominantly unlabeled pool of reagents (e.g., proteins or RNA) spiked only with a small percentage of fluorescently tagged version to allow imaging. This helps avoid the impact of fluorescent tags on phase separation properties.
6) It is important to analyze assays in the context of the time that has elapsed since the condensates first formed. It is now widely appreciated that the material properties of phase-separated condensates change over time. Generally, this manifests as a liquid-to-solid or a liquid-to-gel transition [6]. The rate of change can vary widely depending on the composition of the condensates and buffer conditions.

## Experimental procedures

### Plot of the domain structure, net charge and disorder tendency

Except for the PLD domain, all the other domains were predicted by SMART (http://smart.embl-heidelberg.de/) or NCBI conserved domain (https://www.ncbi.nlm.nih.gov/Structure/cdd/wrpsb.cgi). The PLD was identified using the PLAAC (http://plaac.wi.mit.edu/) [45] The minimal contiguous PLD length for the hidden Markov model was set to 60 and the background frequencies from *Saccharomyces cerevisiae* was set to 100%. The domain structures of the proteins were generated using Illustrator for Biological Sequences. The disorder tendency was predicted by IUPred (http://iupred.enzim.hu/) [46]. To plot the sliding net charge, the sliding window was set to 20 amino acids.

### Liquid-to-solid phase transition assays

The droplets were formed by 7 μM FUS at 75 mM KCl. To generate the scratches, the bottom of the 384-well plate was scratched by the pipette tip. The sample was then added into the well. To generate the air bubbles, the samples were first added into the 384-well plate, and then mixed by pipetting up and down. The air bubbles were generated during the mixing process. Those air bubbles usually stayed in the samples during the whole experimental process. The plate was shaken at 800 rpm at RT. Images were taken with an inverted Olympus IX71 microscope using CoolSNAP HQ camera (Photometrics) and DeltaVision control unit (AppliedPrecision).

### Quantification of phase separation

To quantify phase separation, we plotted the amount of the condensed protein *versus* protein concentration. The amount of the condensed protein was defined by the ratio of I_droplet_ to I_outside_, where I_droplet_ is the integrated intensity inside the droplets and I_outside_ is the integrated intensity outside the droplets.

To segment the droplets, a mask of the droplets was built by thresholding the images and applying a median filter to remove spurious noise detection. The median filter window radius was equal to 2 pixels. The threshold was determined in the same way for each condition as the mean intensity of the background plus *k* times the standard deviation of the background. This way the threshold was well defined even if no droplets were present in the image. The background signal appearing as a sharp peak in the images histogram, background mean, and standard deviation were estimated by the maximum and standard deviation of this peak. The user parameter *k* was set to the value 3 at the beginning of the analysis. The integrated intensity inside the droplet, I_droplet_, and outside, I_outside_ was measured by summing, respectively, the intensity of each pixel inside and outside the droplet mask. If no droplets appeared, the ratio was set to 0. In case of non-zero camera offset, an image could be acquired with shutter closed and its average intensity was removed from each pixel contribution.

## Figures and Tables

**Fig. 5:**
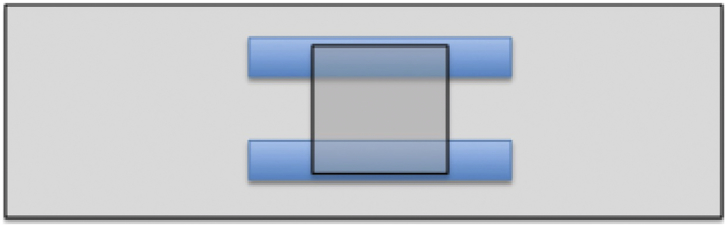
Imaging chamber generated by attaching coverslip to the glass slide using double-sided tape placed parallel to each other, such that a rectangular chamber with two openings on opposite sides is made. The slide and coverslip are shown in gray, and double-sided tapes are shown in blue.

## Appendix A

### A.1 Protocol: PEG silanization of microscopy glass slides and coverslips

*Cleaning coverslips and glass slides*

1. Put in 2% hellmanex for ~ 2 h
2. Rinse with distilled H_2_0
3. Dry with compressed air

*Treatment glass slides/coverslips with polyethylene glycol (PEG)*

1. Add 2 g PEG silane (if PEG solution is ~ 1 mg/ml add 2 ml of solution) to 500 ml toluene
2. Mix thoroughly so PEG does not sink to bottom
3. Add ~ 800 μl concentrated HCl (36% HCl)
4. Put glass slides/coverslips in solution for 18 h at RT and stir continuously (cover the beaker so that there is not loss of liquid)

*Cleaning coverslips and glass slides*

1. Rinse glass slides/coverslips 1 × in toulene
2. Rinse glass slides/coverslips 2 × in ethanol
3. Rinse glass slides/coverslips 2 × in water
4. Dry with compressed air
5. Store with desiccant (if possible)

### A.2 Protocol: Generating lipid-coated imaging chambers

*Materials required*

1) Glass slides (76 × 26 × 1 mm).
2) Coverslips (10 × 10 × 0.17 ± 0.005 mm).
3) Double-sided tape (15 mm wide).
4) Distilled water (ddH_2_O)
5) Ethanol
6) Lens cleaning tissue (GE Healthcare Whatman™).
7) Kimwipes or filter paper
8) Chloroform
9) 1,2-Dioleoyl-sn-glycero-3-phosphocholine (DOPC)
10) Glass test tubes
11) Vortex
12) 1.5-ml Eppendorf tubes
13) Liquid nitrogen or − 20 °C fridge

Step 1 Preparation of imaging chamber
  A) Rinse glass slide and coverslips 1 × with ddH_2_O, 1 × with ethanol, and 1 × with ddH_2_O. Wipe the slides and coverslips dry with lens cleaning tissue.
    Create an imaging chamber by attaching coverslip to the glass slide using double-sided tape placed parallel to each other, such that a rectangular chamber with two openings on opposite sides is made (see Fig. 5).
Step 2 Lipid preparation for coating imaging chamber
  A) Add 25 μl of a 10-mg/ml solution of DOPC lipid in chloroform to the bottom of a glass test tube and allow it to dry for 2 h or overnight.
  B) Resuspend the lipid in 50 μl of an aqueous buffer [generally the same buffer as the one used for carrying out the phase separation assays, e.g., 25 mM Hepes (pH 7.5), 150 mM KCl, 1 mM DTT; hereafter called “assay buffer”] using a vortex. Transfer the lipid suspension to a 1.5-ml Eppendorf tube.
  C) Treat the lipid suspension with three consecutive freeze–thaw cycles using liquid nitrogen or − 20 °C fridge. Transfer the lipid suspension to a glass test tube.
  D) Sonicate the lipid suspension in a water bath at room temperature for 30 min.
  E) Dilute the lipid suspension by adding 250 μl of the assay buffer used in step B. This generates enough material to coat more than 30 imaging chambers.
Step 3 Lipid coating of imaging chamber
  A) Flow in assay buffer into imaging chambers and incubate for 5 min. Generally, 5–10 μl of assay buffer is enough to completely fill the imaging chamber. After incubation, drain out the assay buffer by holding a Kimwipe or filter paper to one of the open ends of the imaging chamber.
  B) Flow in the lipid suspension prepared earlier to completely fill the imaging chamber. Incubate for 45 min–1 h in a humid chamber (e.g., a closed box containing wet filter paper).
  C) Wash the imaging chamber four times or more using assay buffer. Wash by flowing in assay buffer through one of the open ends and draining liquid out from the other open end.
  D) The imaging chamber is now ready to use. In case these chambers are not used immediately, these should be filled with assay buffer and stored in a humid chamber. This helps preserve the integrity of the lipid coating for at least 12–24 h.

*Helpful guidelines and tips*

A) If desired, the stability of the lipid coating could be monitored using fluorescence by doping DOPC lipid with a small fraction of fluorescently labeled lipid molecule.
B) We found the coating protocol works more reproductively when starting from a freshly made solution of lipid (in chloroform) rather than using a lipid solution that has been stored for a while.
